# Systemic Treatment of Metastatic Conjunctival Melanoma

**DOI:** 10.1155/2017/4623964

**Published:** 2017-10-26

**Authors:** Simão Pinto Torres, Teresa André, Emanuel Gouveia, Lívio Costa, Maria José Passos

**Affiliations:** ^1^Department of Medical Oncology, Hospital Professor Doutor Fernando Fonseca E.P.E., 2720-276 Amadora, Portugal; ^2^Department of Medical Oncology, Instituto Português de Oncologia de Lisboa, Francisco Gentil E.P.E., 1099-023 Lisboa, Portugal; ^3^Department of Ophthalmology, Centro Hospitalar Lisboa Central E.P.E., 1150-199 Lisboa, Portugal

## Abstract

Conjunctival melanoma (CM) is an exceptionally rare tumor, with a propensity for local and distant recurrence, with the lungs, skin, liver, and brain being the most common sites of metastasis. Recent progress in systemic treatments, with checkpoint inhibitors and targeted therapies blocking BRAF and MEK, has redefined the standard of care of advanced unresectable and metastatic melanoma. Although most trials did not include patients with conjunctival melanoma, its close molecular and genetic relationship to cutaneous melanoma might suggest a similar response to these novel agents. The authors describe two uncommon cases of metastatic conjunctival melanomas with distinct genetic profiles and, as such, submitted to different systemic treatments.

## 1. Introduction

Conjunctival melanoma (CM) is a rare tumor, with an incidence of 0.02 to 0.08 per 100,000 in white populations. It accounts for 1% to 3% of all ocular malignancies in adults and represents only 1.6% of all noncutaneous melanomas in published series, with increasing incidence in the elderly [1].

CM usually develops within the bulbar conjunctiva, rather than the forniceal or palpebral conjunctiva, and can arise in areas of primary acquired melanosis, from a preexisting nevus or de novo [2].

CM is an aggressive tumor, with a propensity for local and distant recurrence [3].

Pathological features recognized as predictive of poor survival and locoregional recurrence are tumor thickness, nonepibulbar locations, and multifocal CM.

In addition to spreading by lymphatics and the bloodstream, CM can undergo direct extension to the globe and orbit [4].

The most common sites of metastasis are the lungs, skin, liver, and brain.

Although a historically treatment-refractory disease, recent progress in systemic treatments, with important benefits in disease control and survival, has redefined the standard of care of advanced unresectable and metastatic melanoma [5].

## 2. Case Presentation 1 (Patient 1)

A 56-year-old Caucasian woman presented with a pigmented lesion developing on a preexisting pterygium of the right eye, with gradual growth since October 2007. Complete excisional biopsy was performed in March 2010, showing a malignant melanoma of the bulbar conjunctiva, with 1.9 mm in thickness.

The patient was lost to follow-up until July 2013, when she turned to the hospital after developing nasal obstruction and fatigue. Laryngoscopy and head MRI revealed a pedunculated mass on the right oropharyngeal wall, with 31 × 13 mm in diameter.

The mass was surgically removed, with a thorough pathologic assessment revealing an ulcerated metastasis of a conjunctival melanoma, with 6.5 mm in thickness and no subepithelial component.

A staging PET/CT scan showed multiple pharyngeal lymphadenopathies.

The patient was referred to our institution for further management, with the abovementioned findings being confirmed. A mutation in exon 15 of the *BRAF* gene (V600) was detected by Cobas 4800.

**Figure 1:**
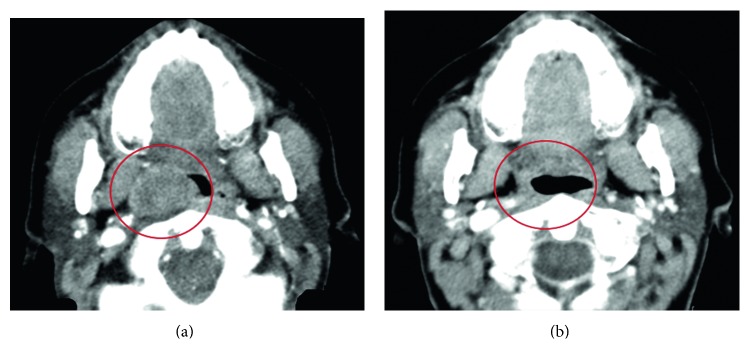
Changes in tumor size in patient 1. A CT scan of the neck, performed at the time of the second recurrence, showing an endophytic mass, with 3.1 × 2.2 cm, on the right oropharyngeal wall, with reduction of its caliber (a). Recent reevaluation showing sustained complete response to vemurafenib, 34 months after starting the treatment (b).

Two months after surgery, there was evidence of local recurrence with symptoms reappearing, and a body PET/CT scan showed a de novo endophytic mass, with 31 × 22 mm, on the previously affected location (Figure 1).

The lesion was considered unresectable, and palliative external radiotherapy, with a total dose of 20 Gy/5 fr, was performed.

In April 2014, systemic therapy was initiated with vemurafenib (960 mg) twice a day.

After one month of therapy, there was full symptomatic resolution.

Restaging evaluation showed sustained favorable response, resulting in complete remission (Figure 1).

There was a need for dose reduction to 480 mg twice a day because of grade 2 arthralgia, grade 2 diarrhea, and grade 1 skin rash.

In February 2017, she was diagnosed with early stage, grade 2, invasive ductal carcinoma of the left breast, pT1cN0snM0, with estrogen receptors +100%, progesterone receptors +1%, HER2 1+, and Ki67 +15%.

She was submitted to a left simple mastectomy with sentinel lymph node biopsy and began hormonal treatment with tamoxifen (20 mg) once a day.

The patient remains free of new lesions as of the last follow-up, on August 8, 2017.

## 3. Case Presentation 2 (Patient 2)

A 51-year-old Caucasian man had a remote history of human immunodeficiency virus (HIV) and hepatitis C virus (HCV) infections, medicated with antiretroviral therapy, with a CD4 count within the normal range and an undetectable viral load. In December 2009, he presented with a hyperpigmented lesion on the temporal conjunctiva of the right eye. Excisional biopsy showed a compound melanocytic nevus.

In December 2011, the lesion recurred on the temporal, inferior, and nasal conjunctiva.

Surgical excision revealed a completely excised malignant melanoma of the bulbar conjunctiva, with 2.4 mm in thickness.

One year later, he was reintervened for a second recurrence on the inferior and temporal conjunctiva.

He was maintained under active surveillance until July 2016, when right cervical adenopathies were noted. Excisional biopsy confirmed metastases of malignant melanoma positive for S100 and MELAN A and negative for CAM 5.2.

The patient was then referred to our institution for further management, with pathologic reevaluation confirming the diagnosis. No mutation in the *BRAF* gene was detected.

A body PET/CT scan documented cervical, submandibular, and pharyngeal adenopathies.

He was submitted to a right cervical lymphadenectomy, with metastases in five of the thirty lymph nodes removed.

Subsequent radiotherapy was initially planned; however, within three weeks, there was rapid disease progression, with multiple de novo subcutaneous nodes of the face and neck.

In December 2016, systemic therapy was initiated with pembrolizumab (2 mg/kg) every 3 weeks.

After the third cycle, there was near to complete resolution of the subcutaneous lesions.

At the last follow-up, on August 23, 2017, the patient was on complete remission, with good tolerance to the ongoing treatment.

## 4. Discussion

We describe two seldom cases of metastatic conjunctival melanomas with distinct genetic profiles and, as such, submitted to different systemic treatments. Both patients have sustained favorable responses up until the writing of this article.

Advances in systemic treatments, with an important enhancement in disease control and survival, have recently changed the standard of care of advanced unresectable and metastatic melanoma [5]. Immunotherapy with checkpoint inhibitors and targeted therapies blocking BRAF and MEK demonstrated significantly improved outcomes compared with conventional therapies [5, 6]. BRAF and MEK inhibitors are indicated for approximately 50% of patients who harbor the *BRAF* V600 mutations, while programmed death-1 (PD-1) inhibitors are effective regardless of *BRAF* mutational status [6–14].

Although most trials did not include patients with conjunctival melanoma, its close molecular and genetic relationship to cutaneous melanoma might suggest a similar response to these novel agents [8]. Griewank et al. [9] identified *BRAF* mutations in 23 of 78 (29%) conjunctival melanomas, a majority of which (91%) were V600E mutations.

In case 1, when the patient began treatment, single-agent therapy with vemurafenib or dabrafenib was the standard of care. Although the combination with BRAF and MEK inhibitors proved to have less toxicity and to be more effective regarding response rates, progression-free survival, and overall survival, it only became available in our country posteriorly [10, 11].

Despite the known risk of the emergence of resistance [12, 13], given the patient's prolonged complete response, the authors opted to maintain single-agent BRAF inhibitor therapy.

Her recent diagnosis of breast cancer raises the question whether the disease can be related to prolonged treatment with vemurafenib.

Secondary tumors, like cutaneous squamous cell carcinomas or keratoacanthoma, can occur in approximately 25% of patients treated with vemurafenib, as a result of paradoxical activation of the mitogen-activated protein kinase pathway in nonmelanoma *BRAF* wild-type cells [14].

Novik et al. [15] reported a relapse of breast cancer after initiating vemurafenib in a patient with *BRAF*-mutated cutaneous melanoma.

At the present time, however, there are insufficient data to make this assumption.

In case 2, there were concerns about the use of a checkpoint inhibitor in a patient with HIV, given there could be immune-related side effects specific to this population.

There is scarce literature regarding this issue. As this is a generalized concern, these patients are usually excluded from trials with immunotherapy.

Davar et al. [16] reported two cases with advanced cutaneous melanoma and concomitant HCV and HIV infections treated with pembrolizumab, with good tolerance and without exacerbation of the underlying infection.

The same was observed in our patient, with treatment proving to be effective and excellently tolerated.

In conclusion, both treatments, with BRAF inhibitors (in the presence of the *BRAF* gene mutation) and PD-1 inhibitors, seem to be valid options in the treatment of metastatic conjunctival melanoma.

Prospective studies are needed to further confirm the efficacy and tolerability of these treatments in this patient population.
